# Improved vessel painting with carbocyanine dye-liposome solution for visualisation of vasculature

**DOI:** 10.1038/s41598-017-09496-4

**Published:** 2017-08-30

**Authors:** Alu Konno, Naoya Matsumoto, Shigetoshi Okazaki

**Affiliations:** 1 Department of Medical Spectroscopy, Preeminent Medical Photonics Education & Research Center, Institute for Medical Photonics Research, Hamamatsu University School of Medicine, Shizuoka, Japan; 2 Central Research Laboratory, Hamamatsu Photonics K.K., Shizuoka, Japan

## Abstract

Vessel painting is one of the most accessible and cost-effective techniques for visualising vasculature by fluorescence microscopy. In this method, the hydrophobic carbocyanine dye DiIC18 labels the plasma membrane via insertion of its alkyl chains into the lipid bilayer. A major disadvantage of this procedure is that it does not stain veins and some microvessels in mouse brain. Furthermore, DiIC18 molecules can aggregate during perfusion, thereby occluding arteries and reducing the success rate and reproducibility of the experiment. To overcome these problems, we developed an improved vessel painting procedure that employs neutral liposomes (NLs) and DiIC12. NLs prevented DiI aggregation under physiological conditions whereas DiIC12 showed enhanced dye incorporation into liposomes and consequently increased staining intensity. Using this method, we successfully labelled all major blood vessel types in the mouse brain, including both veins and microvessels. Thus, liposome-mediated vessel painting is a simple and efficient method for visualising vasculature.

## Introduction

The cardiovascular system is a continuous network that circulates oxygen, nutrients, signalling molecules, and cells and removes metabolic waste. Visualising the vasculature can provide insight into the development and remodelling of blood vessels in normal and pathological states. In mice, major arteries and veins in the brain have been imaged and identified by magnetic resonance imaging and micro-computed tomography^1^. Fluorescence microscopy allows high-resolution and multi-label observation and is thus a useful tool for advancing our knowledge of vascular anatomy and its relationship to other elements. Several methods have been developed to visualise vasculature by fluorescence microscopy^2^, including transgene expression or immunodetection of vascular endothelial markers^3^ and infusion of fluorescent dextrans^4, 5^, labelled lectins^6^, Alexa Fluor 633 hydrazide^7^, or fluorescent resin^8^. These techniques have certain limitations, such as the requirement of transgenic animals (for marker transgene expression), costly reagents (immunohistochemistry, Alexa Fluor 633 hydrazide, and labelled lectins), possible leakage (fluorescent dextrans), and difficulty in perfusing viscous medium (fluorescent resin).

Vessel painting using the lipophilic carbocyanine dye 1,1′-dioctadecyl-3,3,3′,3′-tetramethylindocarbocyanine perchlorate (DiIC18) is a cost-effective and easy means of fluorescently labelling blood vessels^9–11^. However, for reasons that are unclear, not all blood vessel types in mouse brains are adequately labelled by the reported procedure^11, 12^. Furthermore, highly hydrophobic DiIC18 molecules form aggregates during perfusion and occlude microvessels, leading to heterogeneous labelling. These challenges must be overcome in order to clearly detect and identify each element of vasculature.

To this end, we developed an improved vessel painting method using neutral liposomes (NLs) and 1,1′-didodecyl-3,3,3′,3′-tetramethylindocarbocyanine perchlorate (DiIC12) to achieve brighter labelling of all major blood vessel types in mouse brain. The NLs prevented the formation of hydrophobic dye aggregates and occlusion of blood vessels even at physiological salt concentrations. DiIC12 was more efficiently incorporated into NLs and consequently the plasma membrane of endothelial cells lining blood vessels, including veins and microvessels. Our results demonstrate that this procedure is useful for visualising even the smallest vascular elements in the brain.

## Results

### NL and anionic liposome (AL) solutions prevent formation of DiIC18 aggregates at physiological ionic strengths

**Figure 1:**
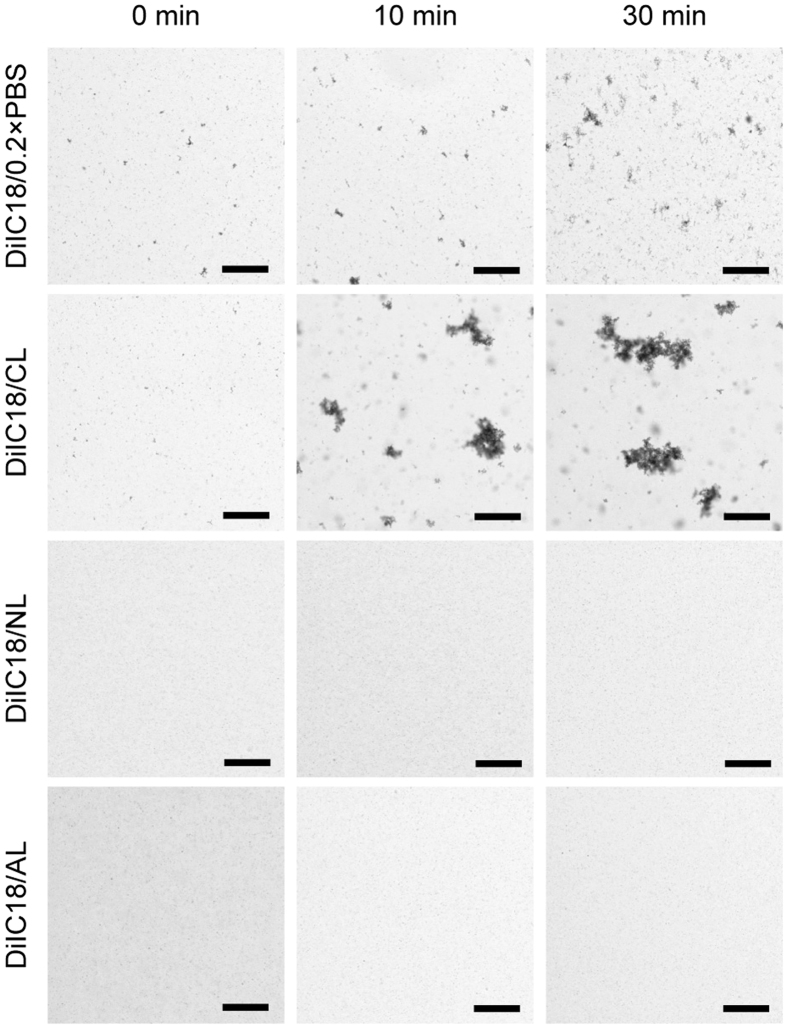
NLs and ALs enable dispersion of DiIC18 at physiological ionic strengths. DiIC18 formed aggregates immediately after preparation of the working solution at low ionic strength (DiIC18/0.2 × PBS). DiIC18/CLs facilitated DiIC18 aggregation, whereas large aggregates did not form in DiIC18/NL or DiIC18/AL solutions for at least 30 min even at a physiological ionic strength. Scale bar = 100 μm.

DiIC18 labels the plasma membrane by inserting its two alkyl chains into the lipid bilayer; however, the highly hydrophobic dye aggregates in the physiological ionic milieu. The original procedure attempted to prevent aggregation by mixing the DiIC18/ethanol stock solution with dilute phosphate-buffered saline (0.2 × PBS; one-fifth of the normal concentration) immediately before use^10^. However, we observed aggregate formation by naked eye within 30 min of preparing the DiIC18 working solution (DiIC18/0.2 × PBS), which we presumed would reduce the reproducibility of the experiment and cause non-homogeneous labelling. We observed the working solution 0, 10, and 30 min after preparation and found that DiIC18 aggregates began forming immediately after addition of DiIC18 and increased in size over time (Fig. 1).

For reproducible perfusion, the formation of large DiIC18 aggregates must be prevented at physiological salt concentrations since the staining solution is exposed to PBS or tissue fluid during the procedure. We speculated that incorporation of DiIC18 molecules into liposomal membranes would lead to their dispersal during vessel painting. To test this possibility, we prepared DiIC18-liposome solutions by mixing DiIC18/ethanol stock solution with cationic liposomes (CL), ALs, or NLs in PBS (50 μM DiIC18, 1 mg/ml liposome in PBS) and evaluated the time course of aggregation (Fig. 1). We found that DiIC18/CL promoted whereas DiIC18/AL and DiIC18/NL prevented the formation of large aggregates for at least 30 min even in PBS (Fig. 1).

### DiIC18/NL solution does not occlude blood vessels during vessel painting

**Figure 2:**
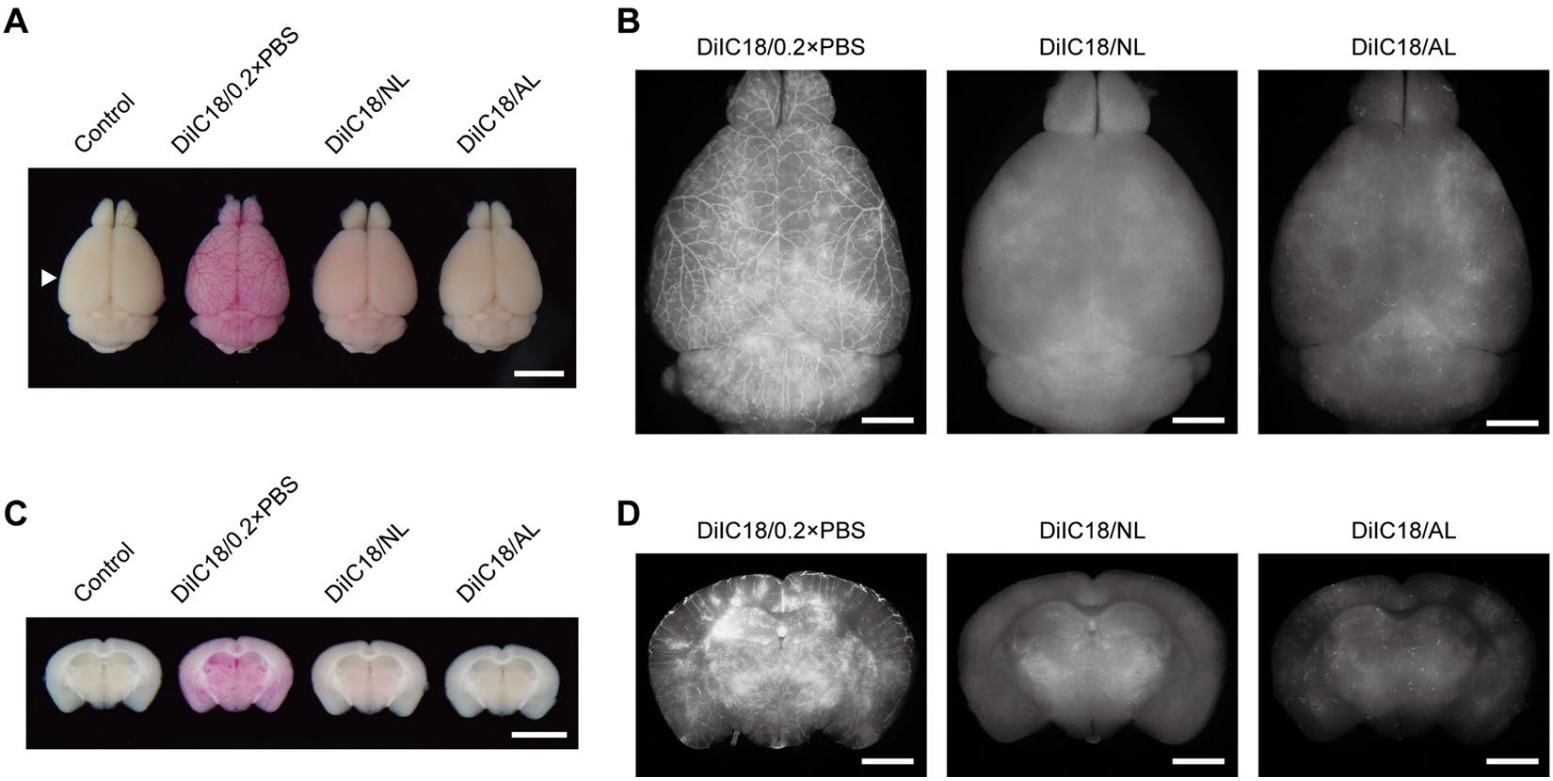
Liposome solutions improve the uniformity of vessel painting. (**A**) Visual examination of vessel-painted brains. The arrowhead indicates the level of sections shown in (**C** and **D**). Scale bar = 5 mm. (**B**) Dorsal view of brains shown in (**A**) under a fluorescence stereomicroscope. Exposure time = 500 ms for DiIC18/0.2 × PBS and DiIC18/NL and 1 s for DiIC18/AL. Note that the colour of the DiIC18/0.2 × PBS-perfused brain is much stronger than that of the other specimens in (**A**), although the fluorescence intensity is only slightly higher than that of the DiIC18/NL-perfused brain. Scale bar = 2 mm. (**C**) Coronal sections of the brains cut at the level indicated by the arrowhead in (**A**). Scale bar = 5 mm. (**D**) Fluorescence microscopy analysis of coronal sections shown in (**C**). Vessel painting with DiIC18/0.2 × PBS produced heterogeneous labelling. Scale bar = 2 mm. Exposure time = 500 ms for DiIC18/0.2 × PBS and DiIC18/NL and 1 s for DiIC18/AL.

**Figure 3:**
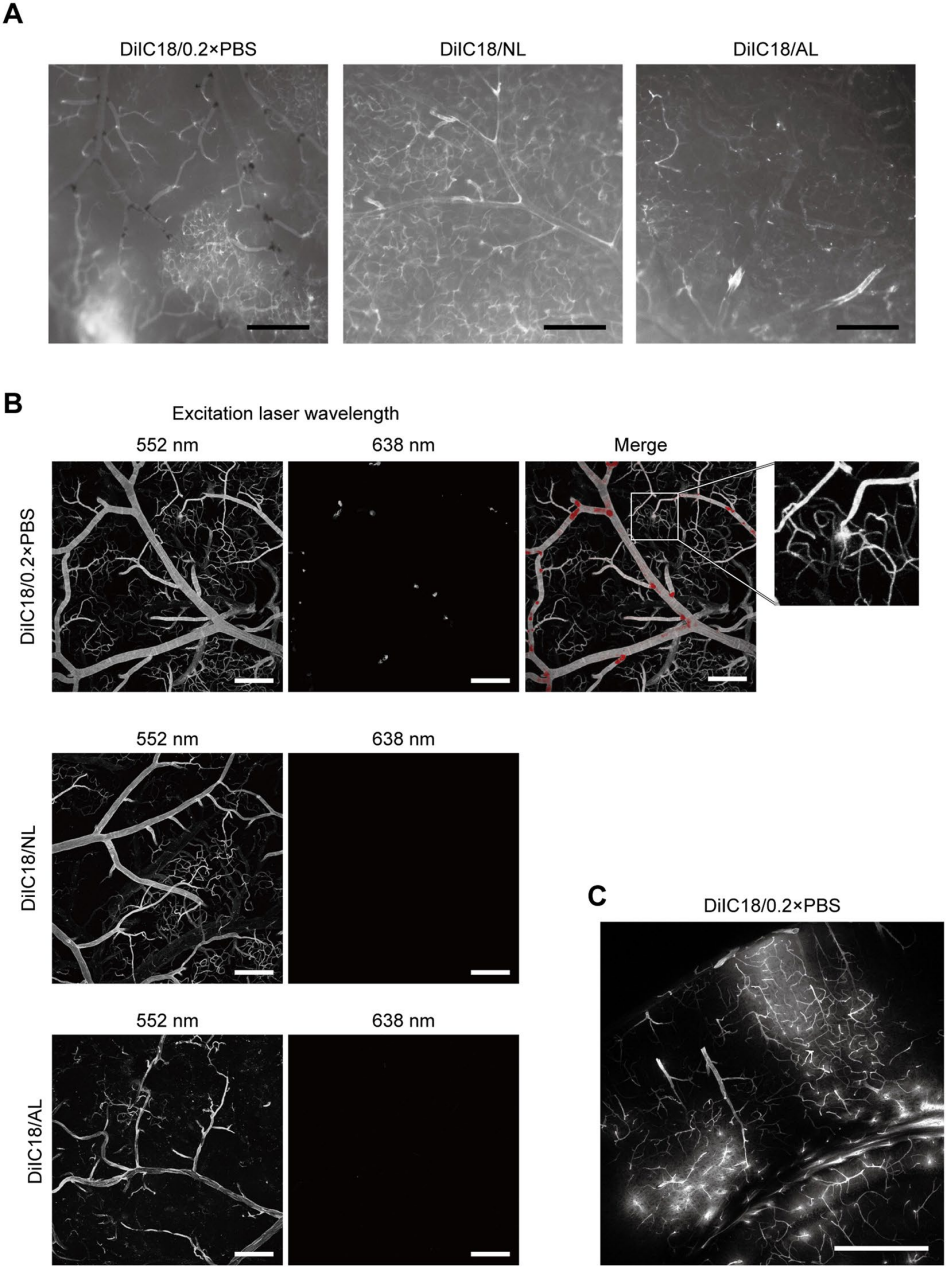
DiIC18 aggregates accumulate at blood vessel branch points. (**A**) Fluorescence stereomicrograph of cerebral surface vessels labelled with DiIC18/0.2 × PBS (left), DiIC18/NL (middle), or DiIC18/AL (right). Dark sediments were present in DiIC18/0.2 × PBS perfused blood vessels, especially at the branch point of arteries. Exposure time = 75 ms (DiIC18/0.2 × PBS), 150 ms (DiIC18/NL), and 300 ms (DiIC18AL). Scale bar = 500 μm. (**B**) Confocal micrograph of cerebral surface vessels. The relative ratio of laser power as 1:1.5:5 for DiIC18/0.2 × PBS, DiIC18/NL, and DiIC18AL. Leakage of DiI is often noted in the brain labelled with DiIC18/0.2 × PBS (inset). Scale bar = 200 μm. (**C**) Confocal microscopy analysis of a coronal section perfused with DiIC18/0.2 × PBS. Scale bar = 500 μm.

Given that DiIC18 was dispersed in AL and NL solutions, we used DiIC18/AL and DiIC18/NL for vessel painting (Figs 2 and 3). Vessel-painted brains showed the strongest staining with DiIC18/0.2 × PBS, and a visual inspection revealed large branches of blood vessels at the cerebral surface. In cases of vessel painting with AL and NL solutions, the colour change of the brains was much less apparent and surface blood vessels were not visible to the naked eye (Fig. 2A). However, under a fluorescence stereomicroscope, the brain stained with DiIC18/0.2 × PBS was not markedly brighter than that stained with DiIC18/NL. In contrast, DiIC18/AL staining was very weak and sparse (Fig. 2B).

We visually inspected coronal sections of vessel-painted brains to assess staining uniformity. The staining pattern of the brain labelled with DiIC18/0.2 × PBS was heterogeneous, with bright pink spots that were inconsistent with the anatomy of the brain; however, no such spots were observed when DiIC18/NL was used (Fig. 2C). The staining patterns of brains labelled with DiIC18/0.2 × PBS and DiIC18/NL differed, as observed under a fluorescence stereomicroscope (Fig. 2D). Overall, DiIC18/NL showed more uniform labelling than DiIC18/0.2 × PBS, although some degree of heterogeneity remained.

Closer observation of the cerebral surface with a fluorescence stereomicroscope revealed the frequent presence of dark red masses that were likely DiIC18 aggregates in blood vessels stained with DiIC18/0.2 × PBS (Fig. 3A). The masses were mostly located at the branch point of arteries. Moreover, the staining pattern with DiIC18/0.2 × PBS was highly heterogeneous: some local microvessel clusters showed an intense signal whereas others had almost none. The staining pattern varied markedly in each experiment (Supplementary Fig. 1). When DiIC18/NL was used, the signal intensity was weaker although the pattern was more homogeneous and no dark masses were observed (Fig. 3A). In addition, vessel painting with DiIC18/NL showed much less variation among experiments (Supplementary Fig. 1). Both DiIC18/0.2 × PBS and DiIC18/NL left some blood vessels, especially veins, unstained or only very weakly stained, as previously reported^11, 12^. Although DiIC18/AL did not aggregate within blood vessels, it had very low signal intensity, except for occasional small regions where a relatively strong signal was detected (Fig. 3A, right).

We examined the cerebral surface by confocal microscopy (Fig. 3B) and found that aggregates at the branch points of blood vessels perfused with DiIC18/0.2 × PBS emitted red fluorescence when illuminated with 638-nm laser. The brains perfused with DiIC18/NL or DiIC18/AL did not exhibit such fluorescence.

Examination of coronal sections of DiIC18/0.2 × PBS-perfused brains revealed bright local staining of the cerebral tissue due to leakage of the dye solution from microvessels (Fig. 3C). In addition, we often observed expansion of lungs and leakage of perfusate from the nose and mouth during DiIC18/0.2 × PBS perfusion, suggesting the rupture of pulmonary capillaries (Supplementary Fig. 1). Such problems did not arise when liposome solution was used. These results indicate that the DiI-NL mixture yields more homogeneous staining and reproducible results.

### Combination of DiIC12 and NL intensely and extensively labels blood vessels

Previous reports^11, 12^ and our results indicate that not all blood vessels in the mouse brain can be labelled by vessel painting. So far, DiIC18 has been used for vessel painting; this dye has two alkyl chains composed of 18 carbons each. Several other DiI variants with shorter alkyl chains are commercially available; we speculated that these would exhibit a lower staining specificity in liposome-mediated vessel painting, and tested DiIC12 in this study.

We compared the incorporation efficiency of DiIC12 and DiIC18 into NLs (Supplementary Fig. 2). The addition of DiIC12 to NL in PBS (DiIC12/NL) produced more fluorescent liposomes, suggesting more efficient dye incorporation. Aggregates were not observed in DiIC12/NL, although fine aggregates were detected in DiIC18/NL with 638 nm laser illumination (Supplementary Fig. 2). This suggests that using DiIC12 can improve the efficiency of liposome-mediated vessel painting.

**Figure 4:**
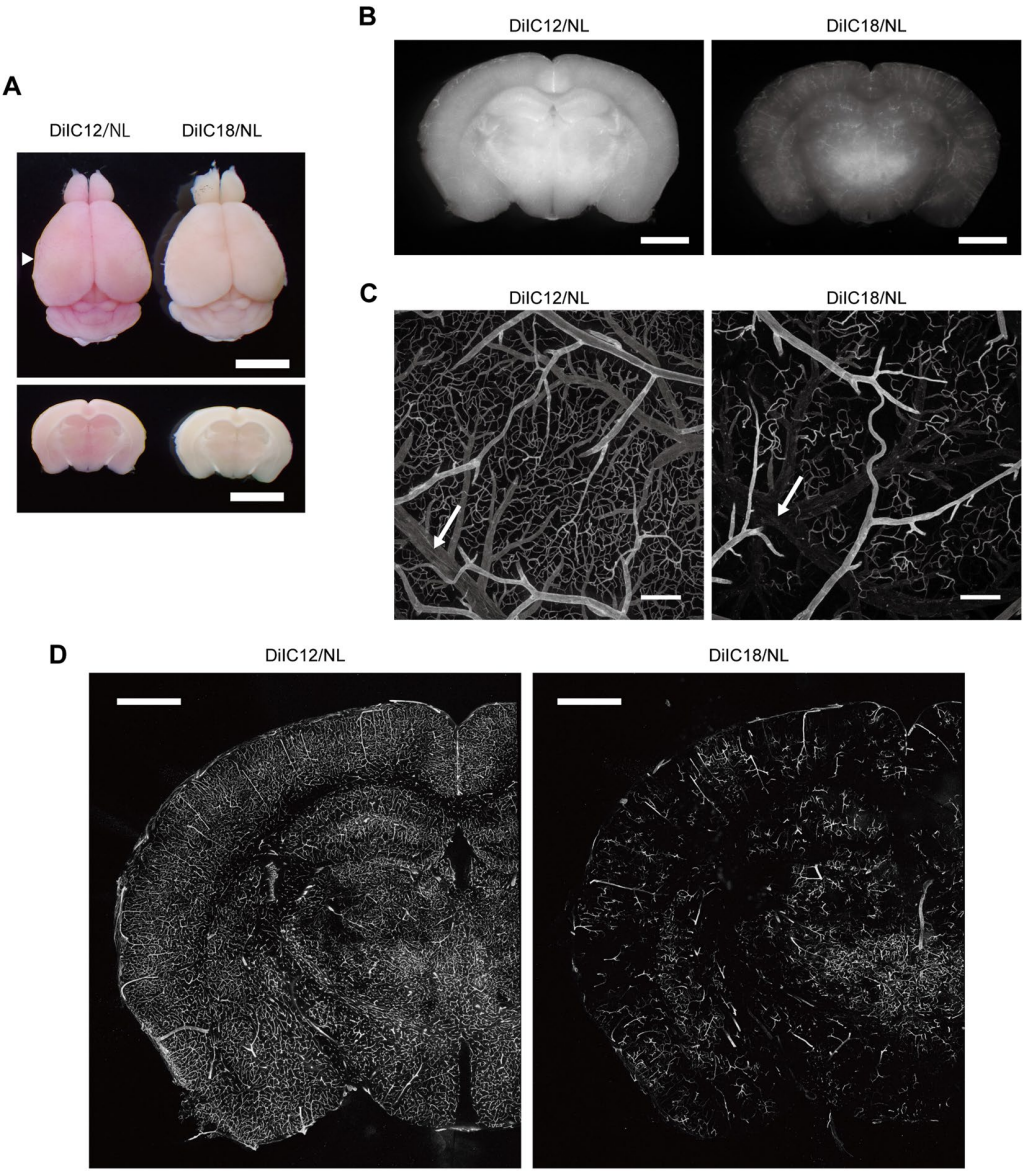
Vessel painting with DiIC12 and NL yields brighter and more uniform labelling of mouse brain vasculature. (**A**) Visual examination of brains perfused with DiIC12/NL or DiIC18/NL. Dorsal view (top) and coronal sections cut at the level of the arrowhead in the top panel (bottom). Scale bar = 5 mm. (**B**) Fluorescence stereoscopy analysis of coronal sections cut at the level of the arrowhead in (**A**). Exposure times are 200 and 400 ms for DiIC12/NL and DiIC18/NL, respectively. Scale bar = 2 mm. (**C**) Confocal microscopy analysis of superficial cerebral blood vessels. The laser power for the DiIC18/NL-painted brain was three times higher than that for the DiIC12/NL-painted brain. Arrows indicate veins. Scale bar = 200 μm. (**D**) Confocal micrographs of coronal sections. The laser power for the DiIC18/NL-painted brain was two times higher than that for the DiIC12/NL-painted brain. Scale bar = 1 mm.

As expected, vessel painting of mouse brains with DiIC12/NL resulted in stronger coloration (Fig. 4A) and fluorescence intensity with more uniform staining (Fig. 4B) than in brains perfused with DiIC18/NL. Among the formulations tested in this study, uniform staining was achieved only when DiIC12/NL was used (Supplementary Fig. 1). Visual inspection under a fluorescence stereomicroscope revealed highly homogeneous labelling with DiIC12/NL in about 50% of specimens.

**Figure 5:**
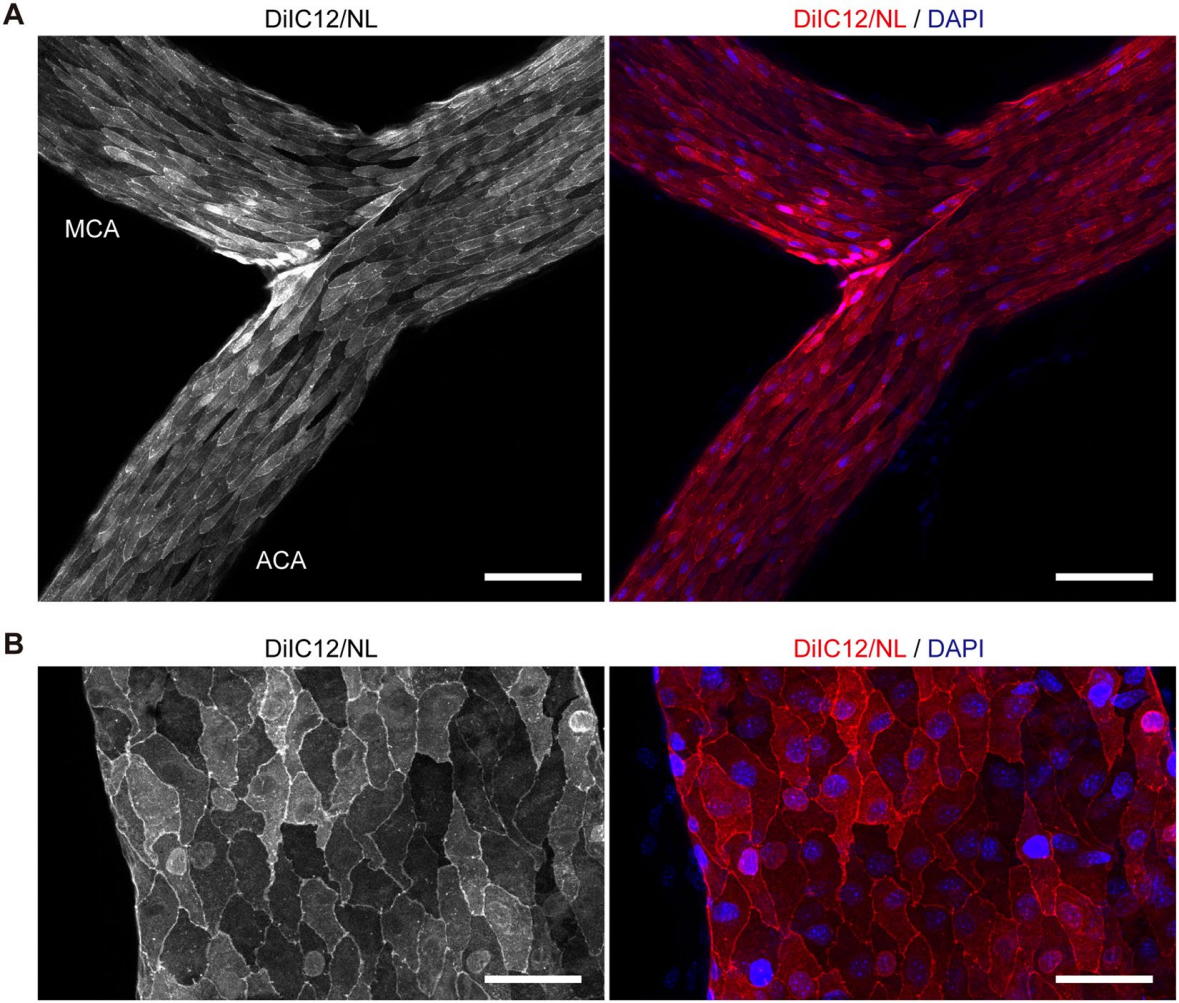
DiIC12-liposome solution delineates endothelial cell contours. (**A**,**B**) Confocal micrograph of a part of the circle of Willis (**A**) and a basal vein (**B**) isolated after perfusion with DiIC12/NL and DAPI. ACA, anterior cerebral artery; MCA, middle cerebral artery. Left panels show the signal from DiI and right panels are merged images of DiI (red) and DAPI (blue) signals. Images show a projection of 12 (**A**) and 15 (**B**) optical sections acquired at 1-μm intervals. Scale bar = 100 μm (**A**) and 50 μm (**B**).

Confocal microscopy analysis of cerebral surface vasculature revealed that DiIC12/NL labelled vasculature more extensively and uniformly than the other formulations tested in this study (Fig. 4C). Importantly, DiIC12/NL labelled veins that were scarcely detectable by DiIC18 staining (Fig. 4C, arrows). Extensive labelling of the vasculature in the coronal section of the hemisphere by DiIC12/NL was also observed by confocal microscopy (Fig. 4D). The signal intensity of DiIC12/NL-perfused vessels persisted for at least for 1 month without obvious reduction upon storage in 4% paraformaldehyde/0.1 M phosphate buffer (pH 7.4) at 4 °C. At higher magnification (Fig. 5), the contours of vascular endothelial cells were distinguishable in DiIC12/NL-perfused brains due to slight variations in staining intensity among cells as well as the bright labelling of cell edges; the nuclei of some cells were also visible even without nuclear staining. The elongated scale-like morphology of endothelial cells lining cerebral arteries were also confirmed by fluorescence immunostaining for VE-cadherin (Supplementary Fig. 3).

## Discussion

In the present study, we improved the vessel painting protocol by introducing NLs and replacing DiIC18 with DiIC12, which produced stronger and more uniform labelling of brain vasculature.

Aggregate formation occurred immediately after dilution of DiIC18 stock solution with 0.2 × PBS and 4% glucose (Fig. 1). Since DiI is less soluble in aqueous solutions with a higher salt concentration, its aggregation may be accelerated during perfusion by exposure to PBS in the tubing used for perfusion and to saline in tissue fluids. Liposome-mediated delivery of fluorescent probes has been tested in cultured cells^13, 14^. Here we investigated whether liposomes can prevent DiI aggregation at physiological salt concentrations. CLs promoted aggregation, likely by repelling positively charged DiI molecules so that they became concentrated outside the physical and electrostatic exclusion volume of liposomes. In contrast, ALs and NLs prevented the formation of large DiIC18 aggregates. This could be useful in other applications where it is not possible to use DiIs under low salt concentrations.

Although a visual inspection suggested that DiIC18/0.2 × PBS labelled the brain much more intensely than DiIC18/NL solution, the difference in signal intensity between them was relatively minor upon fluorescence stereoscopy observation. Since DiIs emit strong fluorescence in the expected spectral range only when they are inserted into a lipid bilayer, the observed colouration of DiIC18/0.2 × PBS-perfused brains could be attributable to DiIC18 molecules that were not inserted into the plasma membrane. DiIC18/AL solution stained blood vessels very weakly and sparsely and was thus inappropriate for vessel painting. This is likely because the surface of the endothelium is covered with a dense layer of negatively charged sialic acids^15^ that would tend to repel ALs and thereby inhibit the fusion of liposomal and endothelial cell membranes.

We found that DiIC18 formed aggregates inside blood vessels and accumulated at the branch points in the absence of liposomes. Major limitations of vessel painting with DiIC18/0.2 × PBS included highly heterogeneous labelling by dye leaked from the local vasculature (Figs 2 and 3) and perfusion failure associated with leakage of the perfusate from the nose and mouth (Supplementary Fig. 1). The most likely cause of these problems is the occlusion and/or narrowing of flow paths by dye aggregates and consequent rupture of weak capillaries by local increases in perfusion pressure. A heterogeneous staining pattern similar to our results was also reported in another study (see Fig. 7 in ref. 16). We also found that the aggregates emitted red fluorescence when excited at 638 nm. Therefore, results of multiple staining using DiIs in conjunction with other dyes with emission maxima in the red region should be interpreted with caution. Consistent with past reports^11, 12^, we found that veins were not strongly labelled by DiIC18/0.2 × PBS. DiIC18/NL solution labelled veins to a slightly greater extent, but this was still insufficient. The degree of microvessel staining was difficult to control with both procedures, *i.e*., some microvessels were strongly stained but others not at all and the patterns varied with each experiment, especially for DiIC18/0.2 × PBS.

As noted above, we found that NLs prevented the formation of large dye aggregates that could occlude blood vessels. However, persistent problems were that the signal intensity of veins was still very weak and that microvessel staining was not highly reproducible. Since the labelling properties of DiIs differ depending on the length of the alkyl chains^17^, we tested the efficiency of vessel painting with DiIC12/NL and found that it provided better results than DiIC18. In addition to more intense labelling overall, DiIC12/NL also stained veins. This is likely because DiIC12 molecules, which are less hydrophobic than DiIC18, would remain soluble long enough to be efficiently incorporated into the liposomal membrane. Furthermore, since DiIs are cationic dyes, more efficient incorporation of DiIC12 molecules would increase the net positive charge of liposomes, which could enhance interactions between liposomal and negatively charged plasma membranes, leading to stronger staining. We found that fine aggregates, which were not noted by bright field observation, were formed in DiIC18/NL working solution (Supplementary Fig. 2). Although the DiIC18 aggregates may be small enough not to occlude major blood vessels, their labelling efficiency is expected to be lower than free or liposome-incorporated molecules. DiIC18 may be as effective as DiIC12 if it is mixed with liposomal lipids in organic solvents, dried, and resuspended in aqueous solution^13^. Nevertheless, the method we describe here is useful due to its simplicity.

As shown in Fig. 4, confocal microscopy analysis of coronal sections labelled by vessel painting with DiIC12 showed a labelling pattern that was comparable to that in an India ink-perfused mouse brain^18^. This indicates that most blood vessel types were labelled by our method, since India ink is a space-occupying material that fills vasculature independent of blood vessel subtypes.

We also found that the contours of endothelial cells could be clearly distinguished by vessel painting (Fig. 5). The staining pattern of major cerebral arteries was also comparable to immunostaining for VE-cadherin (Supplemental Fig. 3) and the endothelial wall imprint observed by corrosion casting^19^. This suggests that the present method is useful for investigating the polarity of endothelial cells without using antibodies. The nuclei of a subset of endothelial cells were also distinguishable as previously reported^10^. This may be partly due to swelling of the membrane above the nuclei of endothelial cells lining large vessels^20^; another possibility is that some free DiI molecules enter cells from the liposomal lumen upon membrane fusion or during perfusion with a fixative that permeabilizes the plasma membrane.

Vessel painting followed by automated analyses for various vascular parameters can be a powerful tool to reveal the features of cerebral vasculature^21^. The application of our improved procedure to this approach would allow the acquisition of a more complete dataset. Furthermore, our liposome-mediated vessel painting method will be especially effective when combined with tissue clearing techniques^22^. Since DiIs label plasma membrane by inserting their carbohydrate chain into the lipid bilayer, they are easily removed by lipid extraction treatments. Therefore, tissue clearing techniques using organic solvents or detergents such as BABB^23^, 3DISCO^24^, CLARITY^25^, or advanced CUBIC^26^ are incompatible with vessel painting. FocusClear has been successfully used to clear brain slices after vessel painting^16, 27^; SeeDB^28^ and Sca*l*eSQ—a detergent-free option of Sca*l*eS^29^—are less expensive alternatives.

## Conclusion

In the present study, we developed an improved liposome-mediated vessel painting technique with NL and DiIC12. This procedure produced higher signal intensity and had lower blood vessel type specificity than the original method, thereby allowing visualisation of virtually all blood vessels in small rodent brains.

## Methods

### Reagents

ALs, CLs, and NLs (Coatsome EL-01-A, EL-01-C, and EL-01-N, respectively) were purchased from Yuka Sangyo (Tokyo, Japan). DiIC18 was purchased from Sigma-Aldrich (St. Louis, MO, USA) (#42364). DiIC12 were purchased from AAT Bioquest (Sunnyvale, CA, USA) (#22035) and Thermo Fisher Scientific (Waltham, MA, USA) (#D383). Syringes, needles, and stopcocks were purchased from Terumo (Tokyo, Japan). Other reagents were purchased from Wako Pure Chemical Industries (Osaka, Japan).

### Animals

8- to 10-week-old male C57BL/6 J mice from Japan SLC (Shizuoka, Japan) were used. All experiments involving mice were performed in accordance with the guidelines and with the approval of the Institutional Animal Care and Use Committee of Hamamatsu University School of Medicine.

### DiI solutions

The original DiI working solution (DiIC18/0.2 × PBS) was prepared immediately before use by adding 200 μl of 6.42 mM DiIC18 in ethanol to 10 ml of 4% glucose in 0.2 × PBS (final DiIC18 concentration: 128.4 μM) and mixing vigorously^10^. For DiI/liposome solutions, 100 μl of 5 mM DiIC18 or DiIC12 in ethanol were added to 10 ml of 1 mg/ml liposome in 1 × PBS (final DiI concentration: 50 μM); the solution was mixed by inverting the tube several times followed by sonication for 1 min.

### Vessel painting

Mice were deeply euthanised with isoflurane and manually perfused via the left ventricle with syringes attached to three-way stopcocks as previously reported^10^. In the original procedure, 2 ml of PBS, 10 ml of DiIC18/0.2 × PBS, and 10 ml of fixative [4% paraformaldehyde/0.1 M phosphate buffer (pH 7.4)] were sequentially injected at a flow rate of 1–2 ml/min. For liposome-mediated vessel painting, we found that omitting the PBS flush had no obvious effect on the result and the faster flow rate was much easier to control by manual injection. Therefore, animals were perfused with 10 ml of DiI/liposome solution followed by 10 ml of fixative at a rate of approximately 5 ml/min. All solutions were used at room temperature. The brain was then isolated and stored in the same fixative solution overnight or longer at 4 °C. For detailed observation of the endothelium, animals were perfused with 10 ml of fixative containing 1 μg/ml of the nuclear stain DAPI following vessel painting. Excess DAPI was washed away by further perfusion with 10 ml DAPI-free fixative to avoid strong staining of the nuclei of non-endothelial cells. After immersion fixation, part of the circle of Willis and a neighbouring basal vein were carefully isolated under a fluorescence stereomicroscope.

### Imaging

Macroscopic images of fixed brains were acquired with an Optio WG-2 digital camera (Pentax, Tokyo, Japan). Whole brain fluorescence images were obtained with a WRAYCAM-SR130M sCMOS camera mounted on an SZX16 stereomicroscope (Olympus, Tokyo, Japan) using WRAYSPECT software (Wraymer, Osaka, Japan). DiI aggregation and DiI-labelled blood vessels were visualised with an SP8 confocal microscope (Leica, Wetzlar, Germany) equipped with HCX PL APO CS 10 × /0.40 DRY and HC PL APO CS2 20 × /0.75 DRY objectives (Leica). Images were obtained with LAS X software (Leica). Membrane-inserted DiI was excited with a 552-nm laser and emitted light was detected within the range of 560–620 nm. DiI aggregates were excited with a 638-nm laser and emission was detected within the range of 650–750 nm. The power of the 638-nm laser was always two times higher than that of the 552-nm laser when both were used. The curved surface of samples made them impossible to image with a defined number of optical sections for z projection; therefore, the power of the excitation laser was set as high as possible at the sample surface so as to avoid signal saturation; samples were imaged to a depth at which the signal was no longer detected while keeping the laser power constant. Raw images were exported from LAS X software in TIF format and processed with ImageJ (National Institutes of Health, Bethesda, MD, USA) and Adobe Photoshop CS6 and Illustrator CS6 (Adobe Systems, San Jose, CA, USA) software. Z projections of optical sections were obtained by maximum projection using ImageJ.

### Immunohistochemistry

After perfusion fixation, a brain was isolated and further fixed at 4 °C. Major cerebral arteries were isolated under a dissection microscope and incubated in 10 mM Tris-HCl (pH 9.0) containing 1 mM EDTA and 0.05% Tween 20 at 60 °C for 20 min for antigen retrieval. It was incubated with 0.5% goat serum in PBST (blocking solution), treated with anti-VE-cadherin antibody (1:100; Abcam, ab33168), and then anti-rabbit IgG antibody Alexa488-conjugate (1:250; Molecular probes, A11008) antibodies and 1 μg/ml DAPI diluted in the blocking solution.

## Electronic supplementary material

Supplementary information
