# Supplementary material for: Improved vessel painting with carbocyanine dye-liposome solution for visualisation of vasculature
Source: Sci Rep. 2017 Aug 30;7:10089. doi: 10.1038/s41598-017-09496-4 (PMC5577039; doi:10.1038/s41598-017-09496-4)
Supplement: Supplementary file 1 — Supplementary information [file 41598_2017_9496_MOESM1_ESM.pdf]

Supplementary Information

Improved vessel painting with carbocyanine dye-liposome solution for visualisation of vasculature

Alu Konno[1], Naoya Matsumoto[2], Shigetoshi Okazaki[1,*]

[1]Department of Medical Spectroscopy, Preeminent Medical Photonics Education & Research Center, Institute for Medical Photonics Research, Hamamatsu University School of Medicine, Shizuoka, Japan

[2]Central Research Laboratory, Hamamatsu Photonics K.K., Shizuoka, Japan

*Correspondence: okazaki@hama-med.ac.jp

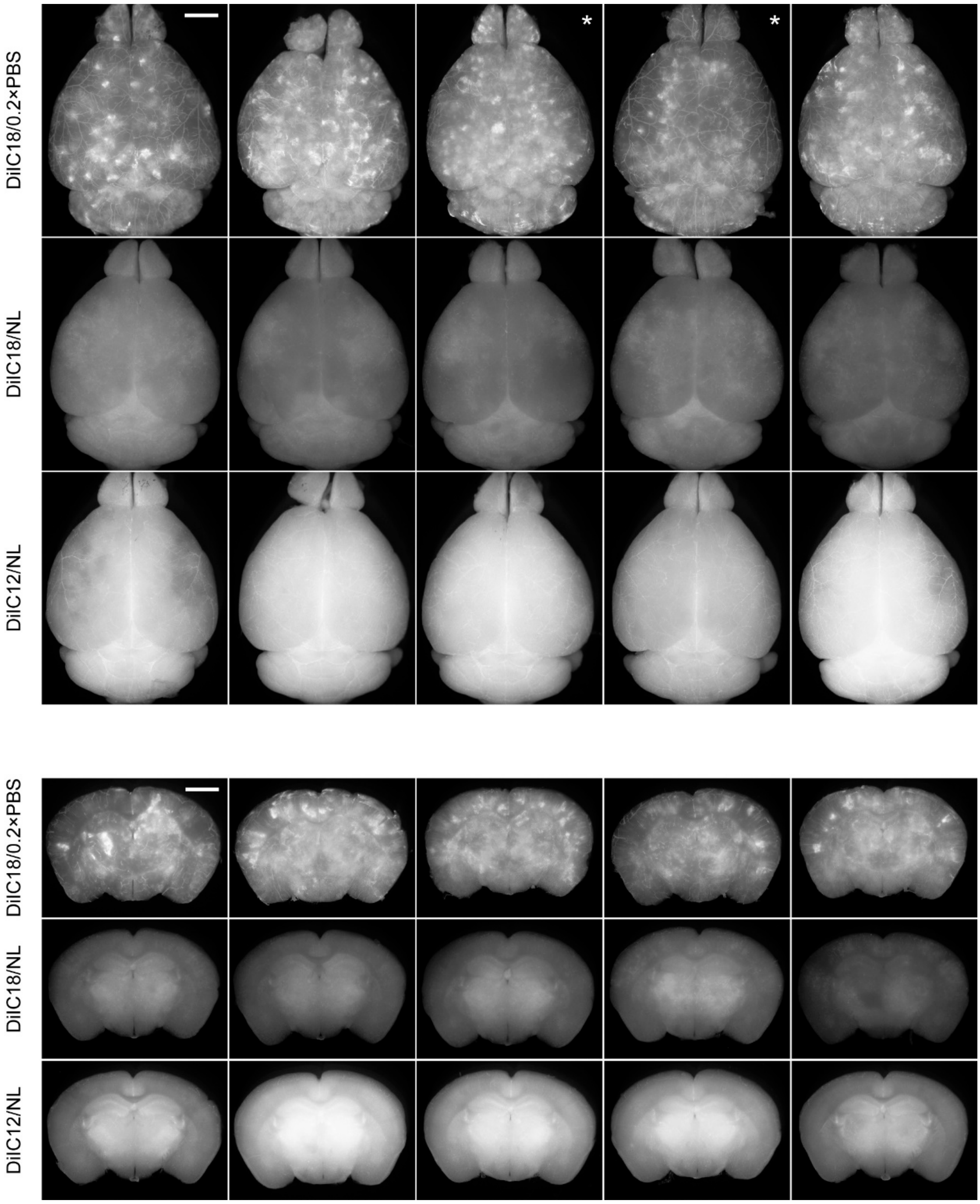

Supplementary Fig. 1. Reproducibility of vessel painting with DiIC18/0.2×PBS, DiIC18/NL, and DiIC12/NL. Top panels show the dorsal view of isolated brains prepared in 15 successive experiments. Asterisks indicate individuals in which there was leakage of perfusate from the airway during vessel painting. Bottom panels show coronal sections of the brains shown in the top panels. Bright local spots in the brains labelled with DiIC18/0.2×PBS are the putative sites of capillary rupture. For all panels, exposure time was 500 ms. Scale bar = 2 mm.

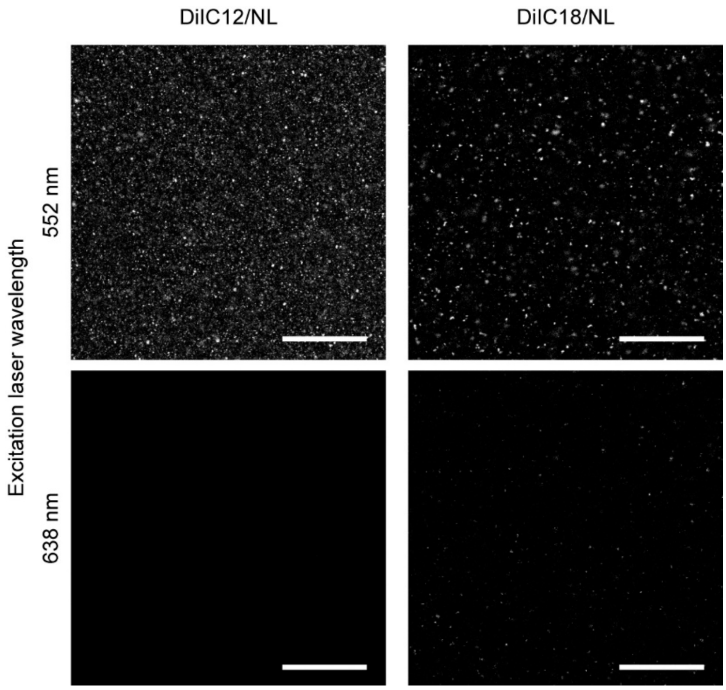

Supplementary Fig. 2. Fluorescence micrograph of DiI12/NL and DiIC18/NL working solutions. Scale bar = 50 μm. Fluorescent liposomes excited by 552 nm laser are more abundant in DiIC12/NL solution. Fine aggregates of DiI molecules excited by 638 nm laser are found in DiIC18/NL solution but not in DiIC12/NL.

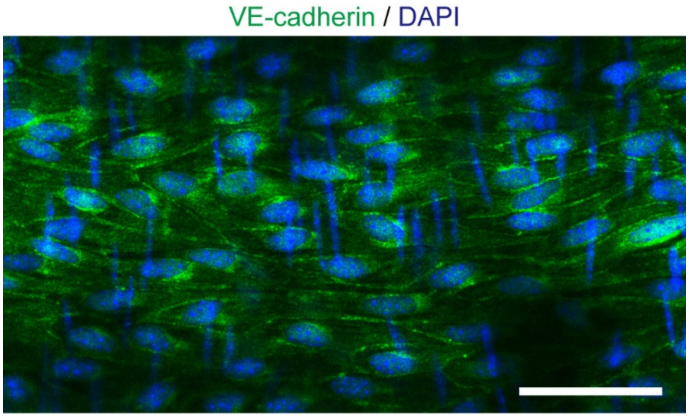

Supplementary Fig. 3. Immunofluorescence microscopy of the endothelium of a middle cerebral artery. VE-cadherin (green) marks the edges of endothelial cells. Elongated out-of-focus signals of DAPI (blue) are the nuclei of smooth muscle cells surrounding the vessel. A projection of five optical sections acquired at 1-μm intervals. Scale bar = 50 μm.
